# Ecosystem carbon stocks of mangroves across broad environmental gradients in West-Central Africa: Global and regional comparisons

**DOI:** 10.1371/journal.pone.0187749

**Published:** 2017-11-13

**Authors:** J. Boone Kauffman, Rupesh K. Bhomia

**Affiliations:** 1 Dept. of Fisheries and Wildlife, Oregon State University, Corvallis, Oregon, United States of America; 2 Center for International Forestry Research (CIFOR), Situgede, Bogor, Indonesia; University of Alabama, UNITED STATES

## Abstract

Globally, it is recognized that blue carbon ecosystems, especially mangroves, often sequester large quantities of carbon and are of interest for inclusion in climate change mitigation strategies. While 19% of the world’s mangroves are in Africa, they are among the least investigated of all blue carbon ecosystems. We quantified total ecosystem carbon stocks in 33 different mangrove stands along the Atlantic coast of West-Central Africa from Senegal to Southern Gabon spanning large gradients of latitude, soil properties, porewater salinity, and precipitation. Mangrove structure ranged from low and dense stands that were <1m in height and >35,000 trees ha^-1^ to tall and open stands >40m in height and <100 ha^-1^. Tremendous variation in ecosystem carbon (C) stocks was measured ranging from 154 to 1,484 Mg C ha^-1^. The mean total ecosystem carbon stock for all mangroves of West-Central Africa was 799 Mg C ha^-1^. Soils comprised an average of 86% of the total carbon stock. The greatest carbon stocks were found in the tall mangroves of Liberia and Gabon North with a mean >1,000 Mg C ha^-1^. The lowest carbon stocks were found in the low mangroves of the semiarid region of Senegal (463 Mg C ha^-1^) and in mangroves on coarse-textured soils in Gabon South (541 Mg C ha^-1^). At the scale of the entirety of West-Central Africa, total ecosystem carbon stocks were poorly correlated to aboveground ecosystem carbon pools, precipitation, latitude and soil salinity (r^2^ = ≤0.07 for all parameters). Based upon a sample of 158 sites from Africa, Asia and Latin America that were sampled in a similar manner to this study, the global mean of carbon stocks for mangroves is 885 Mg C ha^-1^. The ecosystem carbon stocks of mangroves for West-Central Africa are slightly lower than those of Latin America (940 Mg C ha^-1^) and Asia (1049 Mg C ha^-1^) but substantially higher than the default Intergovernmental Panel on Climate Change (IPCC) values for mangroves (511 Mg C ha^-1^). This study provides an improved estimation of default estimates (Tier 1 values) of mangroves for Asia, Latin America, and West Central Africa.

## Introduction

Mangroves provide many ecosystem services that directly benefit biodiversity and human society [1–3]. Mangroves serve as critical habitats for many terrestrial (e.g., bird colonies) and aquatic species (e.g., spawning, feeding and rearing habitats for many fish species; [4, 5]). Mangroves play a critical role in stabilizing sediments and arresting shoreline erosion by trapping particulate matter and binding soil particles [6]. During extreme weather events, mangrove vegetation forms a natural physical barrier against high energy waves and storm surges, thus protecting coastlines and human settlements [7, 8]

Mangroves sequester and store large quantities of carbon (C) [9–11]; and when they are disturbed they shift from important carbon sinks to sources of greenhouse gases (GHG) [12]. As such, they are of great value and interest with respect to climate change adaptation and mitigation strategies [10, 13, 14]. Conservation of mangroves has been increasingly considered as a meaningful way to reduce emissions of GHG in the atmosphere [15–17]. Participation in such mitigation/adaptation strategies requires accurate quantification of existing carbon stocks to meet required monitoring, reporting and verification standards.

Africa hosts about 19% of the world’s mangroves, of which about 20,410 km^2^ (12% of the world’s mangroves and 59% of African mangroves) are located in West-Central Africa [18–19]. These mangroves are important to many people along the Atlantic Coast of Africa. For example, about five million people are dependent on small-scale fisheries for their livelihoods and fish are a major source of dietary protein [18]. Threats on existing mangroves includes increasing human pressures resulting from deforestation, land cover change, upstream diversions of freshwater, and pollution [18].

Few studies have examined ecosystem carbon stocks of African mangroves and especially along the Atlantic Coast of West-Central Africa. Given the ecological and economic importance of mangroves and the fast growing coastal human population in this region of Africa, studies are needed to collect information that will inform policy decisions to reconcile economic development with mangrove conservation. The objectives of this study were to quantify total ecosystem carbon stocks in mangrove stands occurring in four different locations along the Atlantic coast of West-Central Africa. We determined the ecosystem carbon stocks of mangroves across broad gradients of latitude, climate, salinity, soil characteristics, and ecosystem structure. We hypothesized that ecosystem carbon stocks would be related to differences in precipitation, soil salinity, soil pH, and aboveground forest structure at both local and regional scales. A second objective of this study was to compare the ecosystem carbon stocks of West-Central African mangroves to those of other locations throughout the world. To accomplish this we assembled a global data set comprised of mangrove ecosystem carbon stocks that were determined in a similar manner to this study. We also compared this global data set to the default values for mangrove carbon stocks provided by the Intergovernmental Panel on Climate Change (IPCC) [17].

## Study areas

**Fig 1:**
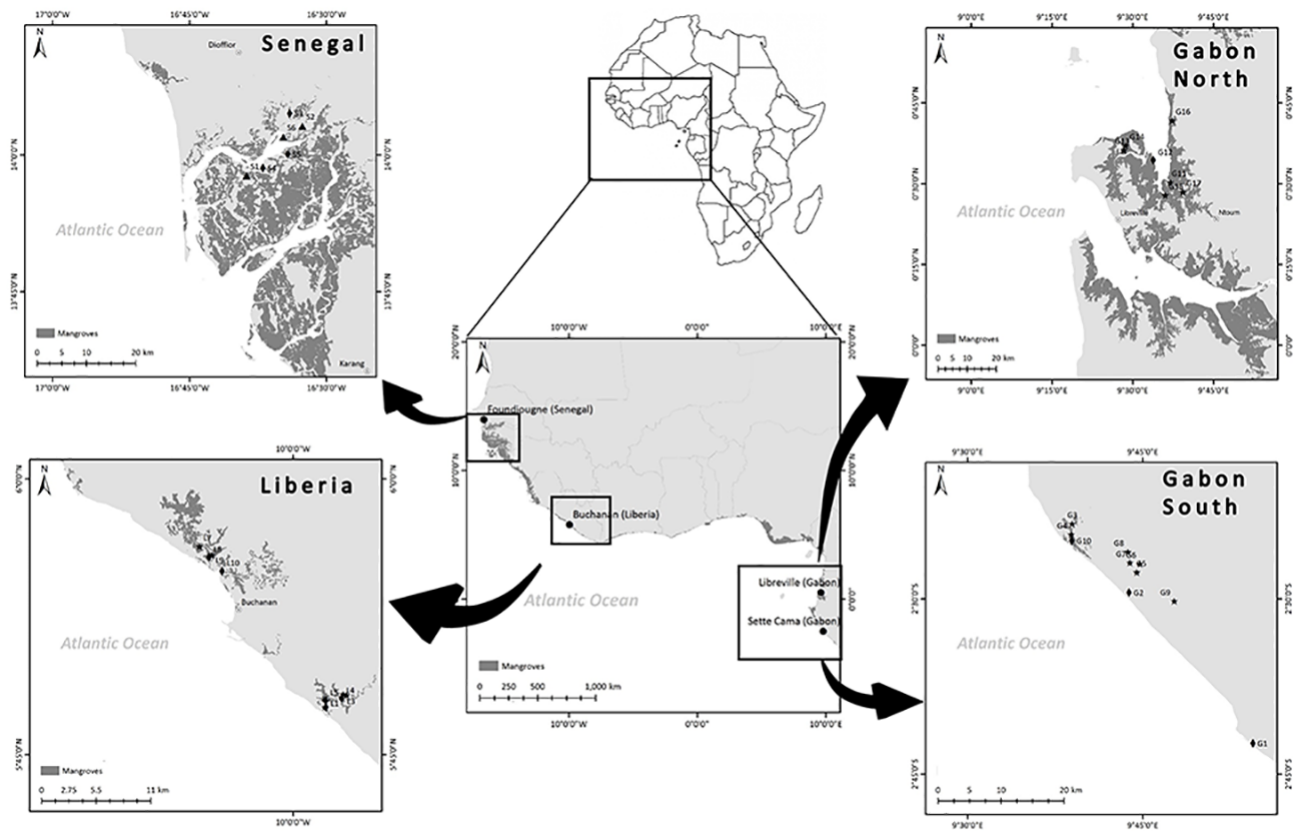
Study area and sampling locations across three West African countries—Liberia, Senegal and Gabon. Mangroves were sampled along the Cess and Mechlin (St Johns) Rivers in Liberia, in the Saloum Delta, Senegal, Mondah Bay, Akanda National Park, (Gabon North) and the Ndougou Lagoon (Gabon South).

We sampled 33 mangroves in 4 regions of 3 countries along the Atlantic coast of West-Central Africa (Fig 1). The general regions we sampled included: (1) the Saloum Delta, Senegal (n = 6); (2) the Cess and Mechlin (St Johns) Rivers, Liberia (n = 10); (3) within, or near Akanda National Park, Mondah Bay, Northern Gabon (n = 7); and, (4) the Ndougou Lagoon, Southern Gabon (n = 10; Fig 1). Along this section of the African Atlantic coast, climate varies from arid and semi-arid landscapes in the north, to humid wet tropical conditions in the equatorial regions (Table 1). The soils of West-Central African mangroves are quite varied reflecting a diversity of geologic and pedogenetic processes. Soils in our study sites were composed of quartzitic sands (in southern Gabon) to finer textures ranging from silty clay to sandy silts in the other sites [20].

**Table 1:** The approximate latitude of sampled mangroves, mean annual temperature, mean annual precipitation, köppen classification of landscapes encompassing the study sites [21], and soil porewater characteristics and soil depth of sampled mangroves. Climatic data are from http://en.climate-data.org/continent/africa/. Site specific information can be found in S1 Table.

Location	Number of sites	Approximate latitude	Ppt. (mm)	Temp (°C)	Classification	Soil pH	Soil porewater salinity (ppt)		Soil depth (cm)	
						Mean ± SE	Range	Mean ± SE	Range	Mean ± SE
**Senegal**	6	N 14°00'	650	23.1	Arid steppe-hot (Bsh)	6.5 ± 0.1	46–96	64 ± 9	173–>300	254 ± 16
**Liberia**	10	N 5°51'	3346	25.9	Tropical monsoon (Am)	6.9 ± 0.2	5–35	16 ± 3	90–>300	231 ± 16
**Gabon North**	7	N 00°34'	2883	26.2	Tropical monsoon (Am)	7.1 ± 0.1	17–40	30 ± 3	>300	>300
**Gabon South**	10	S 2°27'	1818	25.5	Tropical savanna (Aw)	6.5 ± 0.1	5–42	24 ± 4	182–>300	248 ± 13

## Materials and methods

We received all necessary permits and permission to conduct studies from Parcs Gabon and the Liberian Ministry of Agriculture. Additional permission to sample was granted from the communities of Mounde, Ndjirnda, Fambine, Diamniadior, and Baouth Senegal; Barcoline and Edina Liberia; and Sette Cama, Gabon. This entailed getting permission from private land owners who willingly provided this permission

The dominant mangrove trees encountered during our studies included *Rhizophora mangle L*, (Rhizophoraceae), *Rhizophora racemosa L*. (Rhizophoraceae) and *Avicennia germinans* (L.) Stearn (Acanthaceae). In addition, we also encountered *Laguncularia racemosa* (L.) C. F. Gaertn. (Combretaceae), *Conocarpus erectus L*. (Combretaceae), and *Avicennia africana* Palisot de Beauvois (Acanthaceae). Mangroves were separated into structural/geomorphic classes similar to those defined in references [22–24]. These classes are: a) tall mangroves with a mean height > 10 m and usually occurring on the margins of rivers and estuaries; b) medium mangroves that form dense stands of trees of 3 to 10 m in height, usually as interior forest environments in areas of higher precipitation (Liberia and Gabon) and on estuarine margins in semiarid environments (Senegal); and c) low mangroves, composed of dense stands of trees whose heights are < 3 m and occurring inland of riverine margins and ecotonal to upland ecosystems in semiarid environments.

The mangroves of the Soloume Delta, Senegal consisted of medium stands along the estuary fringe while low mangroves dominated in the interior and upland edge of mangrove environments. Medium mangroves were dominated by *R*. *racemosa* while low mangroves were dominated by *R*. *mangle*, *A*. *germinans* and *A*. *africana*. In Liberia, tall mangroves occurred on estuarine margins and medium mangroves in the interior and upland margins. All were dominated by *R*. *racemosa*. In both Northern and Southern Gabon, the majority of mangroves were tall in stature and dominated by *R*. *racemosa* except for 3 of the Southern Gabon sites that were dominated or co-dominated by *A*. *germinans*. Of the 17 sampled Gabon sites, only three were medium in stature (and dominated by *R*. *racemosa*).

### Field sampling

The composition, structure, and ecosystem carbon stocks of the mangrove sites were measured following methods outlined by Kauffman and Donato [25]. At each site, we collected data necessary to determine species composition, tree density, basal area, and total carbon stocks. Total carbon stocks were derived from measurements of above and belowground tree biomass, downed wood (dead wood on forest floor) and soils to the depths of the marine sands or bedrock. Our scope of inference was defined as all aboveground carbon pools plus belowground organic carbon to a maximum depth of 3 m; therefore our carbon storage estimates are conservative in cases where organic soil depth exceeded 3 m.

### Biomass of trees and shrubs

At each sampled mangrove stand, six plots were established 20 m apart along a randomly established 100 m transect. All components necessary to determine ecosystem stocks were collected in each of the 6 plots.

Composition, tree density and basal area were quantified through identification of the species and measurement of the mainstem diameters of all trees rooted within each plot. The plot size for trees >5 cm in diameter was 154 m^2^ (7 m radius). Trees <5 cm in diameter were measured in a nested plot of 12.6 m^2^ (2 m radius). The diameter of *R*. *racemosa* and *R*. *mangle* trees was measured above the highest prop root. Other mangrove species were measured at 1.3 m above the soil surface.

Allometric equations were used to calculate tree biomass for each site. We used genus specific formulas for the *Rhizophora* and *Avicennia* spp [26]. Belowground root biomass for mangrove trees was calculated using a general mangrove equation [27]. Tree carbon was calculated by multiplying biomass by a factor of 0.48 for aboveground and 0.39 for belowground biomass [25].

Standing dead trees were included in aboveground biomass calculations. Dead trees were separated into three classes depending on the existing branches and twigs attached to the dead tree at the time of sampling. Class I represented a recently dead tree with the majority of primary and secondary branches still attached to the tree. Class II dead trees had primary branches but had lost their leaves and finer secondary branches. Class III dead trees only had the main trunk (all branches lost). Biomass of class I dead trees was estimated to be 97.5% of a live tree, class II—80% of a live tree, and class III—50% of a live tree. Dead aboveground vegetation biomass was converted to carbon mass using conversion ratio of 0.47 [25]. Standing dead trees in the low forests were very rare and were included with live trees, if present.

### Downed wood

We used the planar intersect technique adapted for mangroves to calculate mass of dead and downed wood [25, 28]. At the center of each plot, four 14 m transects were established. The first was established in a direction that was offset 45° from the azimuth of the main transect. The other three were established 90° clockwise from the first transect. At each transect, the diameter of any downed, dead woody material (branches, prop roots or mainstems) intersecting the transect was measured. Wood debris ≥2.5 cm but <7.5 cm in diameter at the point of intersection was measured along the last 5 m of each transect. Wood debris ≥7.5 cm in diameter at the point of intersection was counted from the second meter to the end of the transect (12 m in total). Large downed wood was separated in two decay categories: sound and rotten. Wood was considered rotten if it visually appeared decomposed and broke apart when kicked. To determine mass, we used data of specific gravity of downed wood determined for mangroves of the same genera [22]. Downed wood was converted to carbon using a factor of 0.50 [25]. Mangrove ecosystems generally have no or very little litter; therefore it was not measured [17, 29, 30].

### Soil carbon and nutrients

At each of the six plots in each sampled stand, soil samples were collected to determine bulk density and nutrient concentration. This was accomplished by extracting a soil core with a peat auger consisting of a semi-cylindrical chamber with a 22.95 cm^2^ cross sectional area. This auger was efficient for collecting relatively undisturbed cores from wet soils under mangroves [10, 25]. The core was systematically divided into depth intervals of 0–15 cm, 15–30 cm, 30–50 cm, 50–100 cm and >100 cm (if parent materials were not encountered before 100 cm in depth). At each depth, samples of a known volume were collected. These were then transported to the laboratory, dried to constant mass, and then weighed to determine bulk density. At each sampling point, we measured soil depth by inserting a graduated aluminum probe until refusal (rock/marine sands) at three locations near the center of each plot. The probe length was ≈3 m which is the inference limit of study when mangrove soils exceeded 3m in depth.

We sampled interstitial salinity and pH of the ground water collected in the bore holes using methods described in [31, 32]. A portable handheld refractometer (VEE GEE STX-3, range—0–100 parts per thousand) and pH meter (Milwaukee Instruments, Inc., pH56, pH -Temperature meter) were used for measuring salinity and pH of the soil pore water. Care was taken to ensure that no surface water mixed with the sampled soil porewater as surface water was usually lower in salinity. Porewater was sampled at each soil sampling plot (n = 6 in each sampled stand).

In the laboratory, the concentration of carbon and nitrogen were determined using the dry combustion method (induction furnace). Mangrove soils from Senegal and Liberia were analyzed by using Thermo Flash EA 1112 series C-N Soil Analyzer at the Seagrass analytical lab, Florida International University, Miami, USA. Soil samples from Gabon were analyzed using a Costech EA C-N Analyzer at University of Hawaii-Hilo analytical laboratory. Bulk density and carbon concentration were combined with plot-specific soil depth measurements to determine the soil carbon stocks.

Total ecosystem carbon stocks were defined at the sum of the aboveground carbon pools (consisting of trees, and downed wood) and belowground stocks (consisting of soils and roots). Differences in basal area, tree density and ecosystem carbon stocks among different regions (Senegal. Liberia, Gabon North and Gabon South) and mangrove types (low, medium and tall) were separately tested with one-way analysis of variation (ANOVA). Differences between the size classes within regions and the same size classes between regions were also tested using an ANOVA. When parameters did not conform to the assumptions of parametric statistics, non-parametric analyses (Mann-Whitney tests) were employed. Relationships of environmental parameters and carbon stocks were tested via simple regression analysis. Data are reported as mean ± one standard error.

### Global comparisons

To compare West-Central mangroves to the ecosystem carbon stocks of mangroves throughout the world, we compiled data of mangroves sampled in a similar manner from Latin America (Eastern Pacific and Western Atlantic Oceans), the Asia-Pacific (Western Pacific and Indian Oceans), and the Arabian/Oman Gulf (Northern Indian Ocean). We limited this analysis to those studies which followed the same methods for quantification of ecosystem carbon stocks as described above [25]. This was limited to those studies that included either the entire soil profile or to a default depth of ≈3m in deep estuarine/deltaic sediments. A total of 158 stands were included in this analysis.

## Results

### Vegetation composition and structure

**Fig 2:**
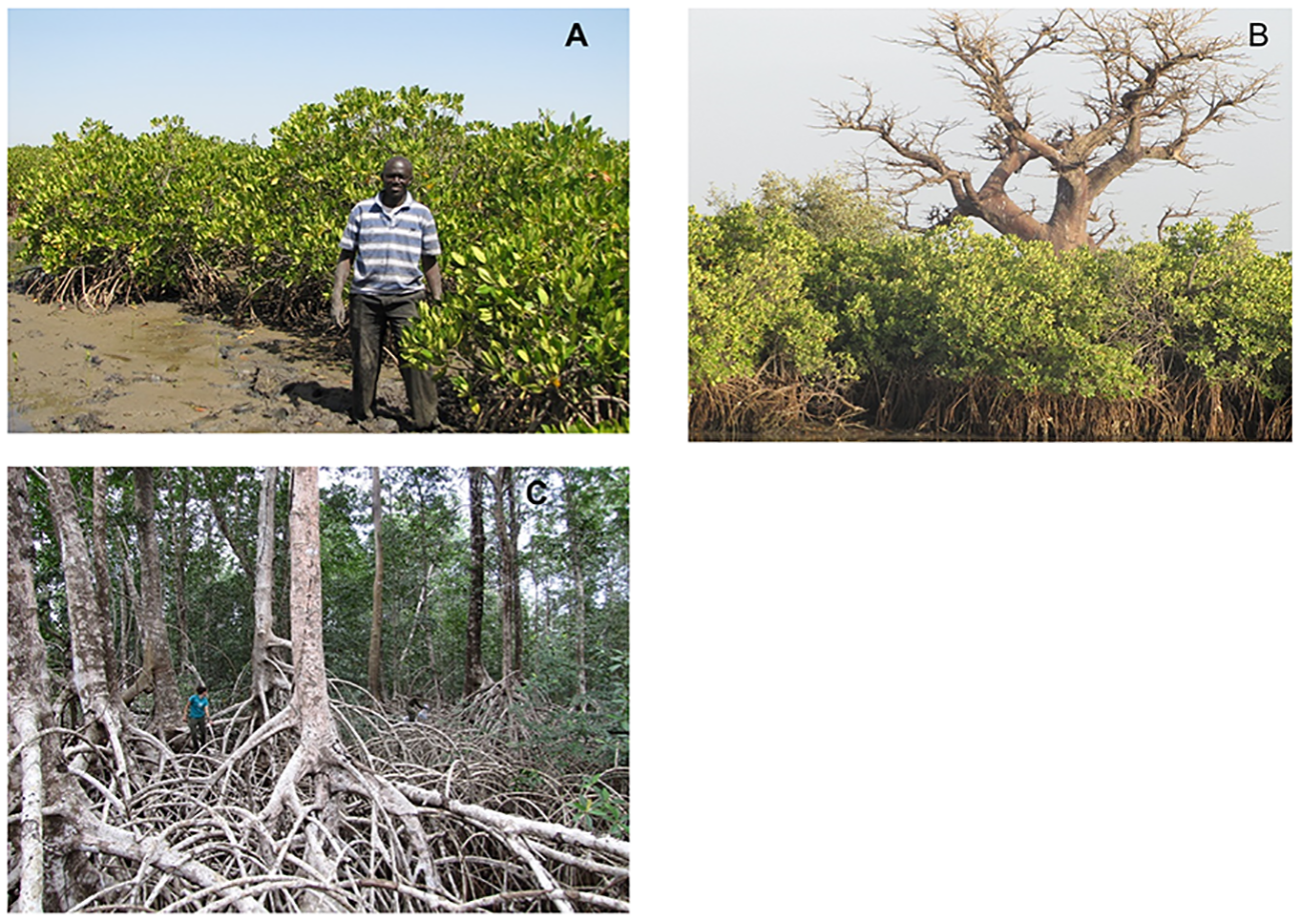
A: A low mangrove plant community in the Saloum Delta, Senegal. B. Medium mangrove of the Saloum Delta, Senegal. The baobab tree in the background is located on uplands behind the mangrove. C. A tall mangrove, of the Ndougou Lagoon, Gabon. The person in the foreground is about 3 m above the soil surface.

There was tremendous variation in the composition, structure and ecosystem carbon stocks of the mangroves occupying the estuarine tidal zones of the Atlantic coast of Africa. The mangroves ranged from dense low stands <1m in height and dominated by *Avicennia* spp to stands >40m height and dominated by *R*. *racemosa* (Fig 2). Compositional and structural differences were predicted given the great variation in latitude, climate, soils and hydrological characteristics of the mangroves (Table 1). Sample sites were distributed from 14° N in Senegal to the equator (0–2° S) in Gabon. Annual precipitation of the study sites ranged from 650 mm to 3346 mm. Salinity of soil porewater ranged from <5 ppt in some of the tall mangroves of Gabon South to 96 ppt in the interior low mangroves of Senegal. The soils ranged in depth from 90 cm to ≥300 cm.

**Fig 3:**
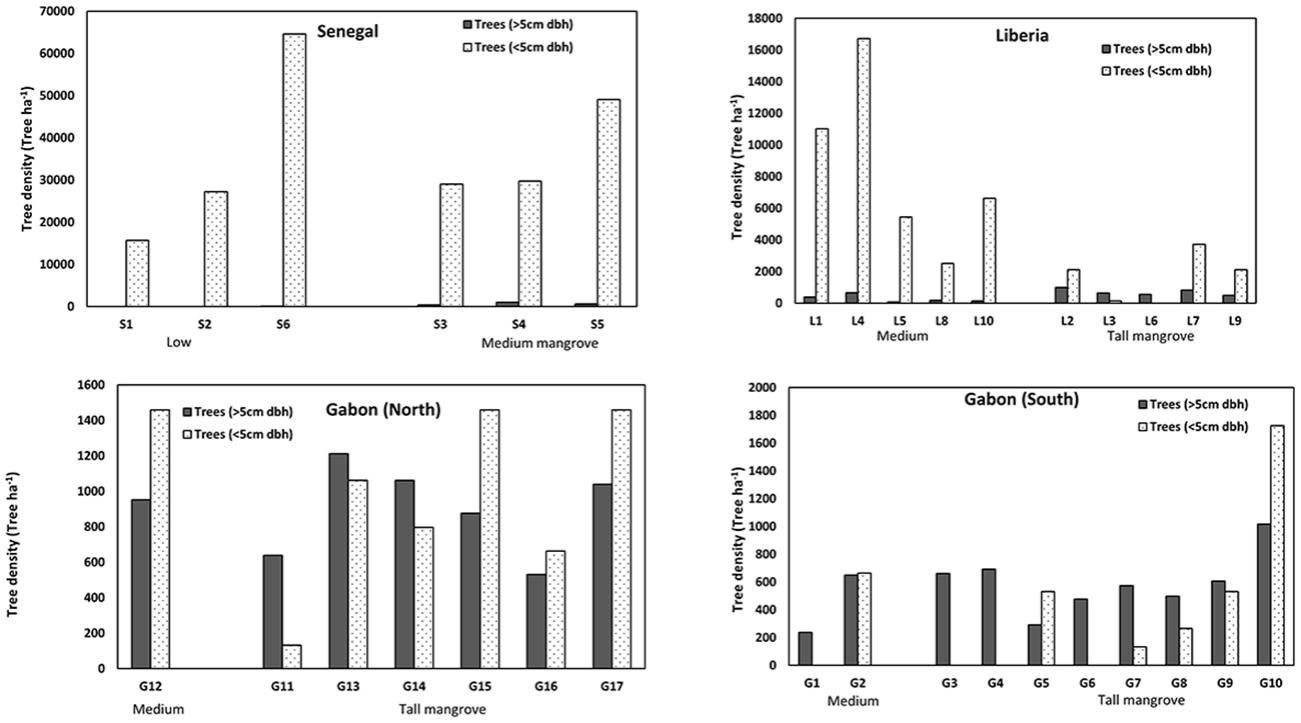
Mangrove tree density (trees ha^-1^) in Liberia (A), Senegal (B), Gabon North (C) and Gabon South (D).

Mean tree density in the mangroves varied significantly among the 4 regions ranging from ≤1000 trees ha^-1^ in Gabon South to >35,000 ha^-1^ in Senegal (p = 0.05; Fig 3). We also found differences in density of the same structural/geomorphic classifications when testing between the 4 regions. For example, the mean density of tall mangroves ranged from 1000 ha^-1^ in Gabon South to 2318 ha^-1^ in Liberia (p = 0.10). Tree density tended to be higher in low and medium mangroves. For the medium mangroves, density ranged from 8752 ha^-1^ in Liberia to 36,605 ha^-1^ in Senegal (p = 0.04). Density in low mangroves of Senegal was as high as 64,639 ha^-1^. The majority of trees in low and medium stands were <5cm in diameter.

**Fig 4:**
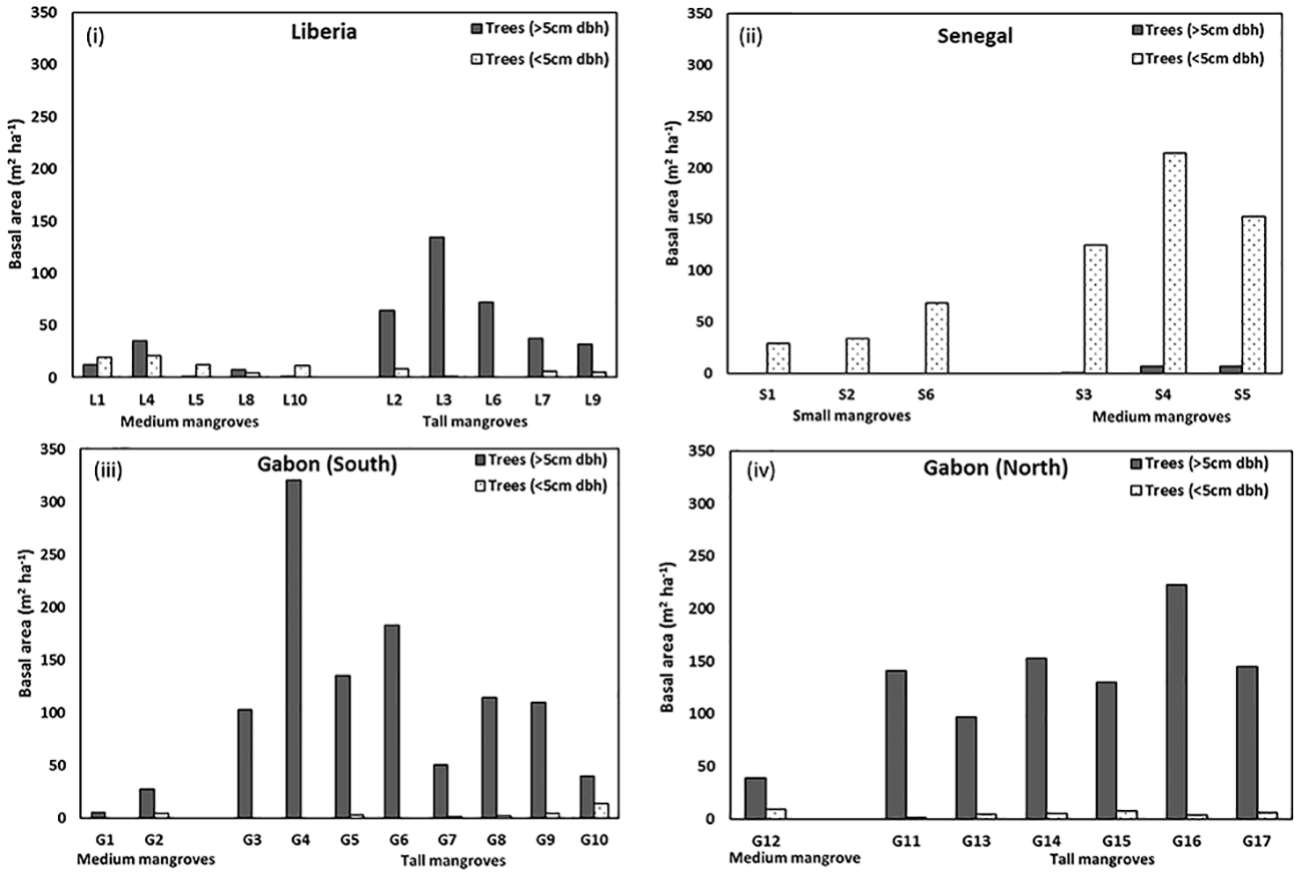
Mangrove tree basal area (m^2^ ha^-1^) in Liberia (i), Senegal (ii), Gabon South (iii) and Gabon North (iv).

The variation in structural diversity of mangroves of West-Central Africa is reflected in the broad range in basal area of individual stands (0.8 m^2^ ha^-1^ to 53.3 m^2^ ha^—1^; Fig 4). Basal area is a good metric that captures tree density and the size of the trees at a site. The areas with the largest basal area were the medium mangroves of Senegal (32.1 m^2^ ha^-1^) and tall mangroves of Gabon North (25.4 m^2^ ha^-1^; Fig 4). The high density of relatively small trees in Senegal medium mangroves resulted in a higher basal area than tall forests with large trees but at lower densities. At each of the 4 regions, the larger statured forests had greater basal areas than smaller-statured forests. For example, the mean basal area of low and medium mangroves was 7.4 and 32.1 m^2^ ha^-1^ in Senegal (p = 0.04); and in Liberia the mean basal area of medium and tall mangroves was 4.1 and 11.7 M^2^ ha^-1^, respectively (p = 0.06). The mean basal area of tall mangroves ranged from 11.7 m^2^ ha^-1^ in Liberia to 25.4 m^2^ ha^-1^ in Gabon North (p = 0.008).

**Fig 5:**
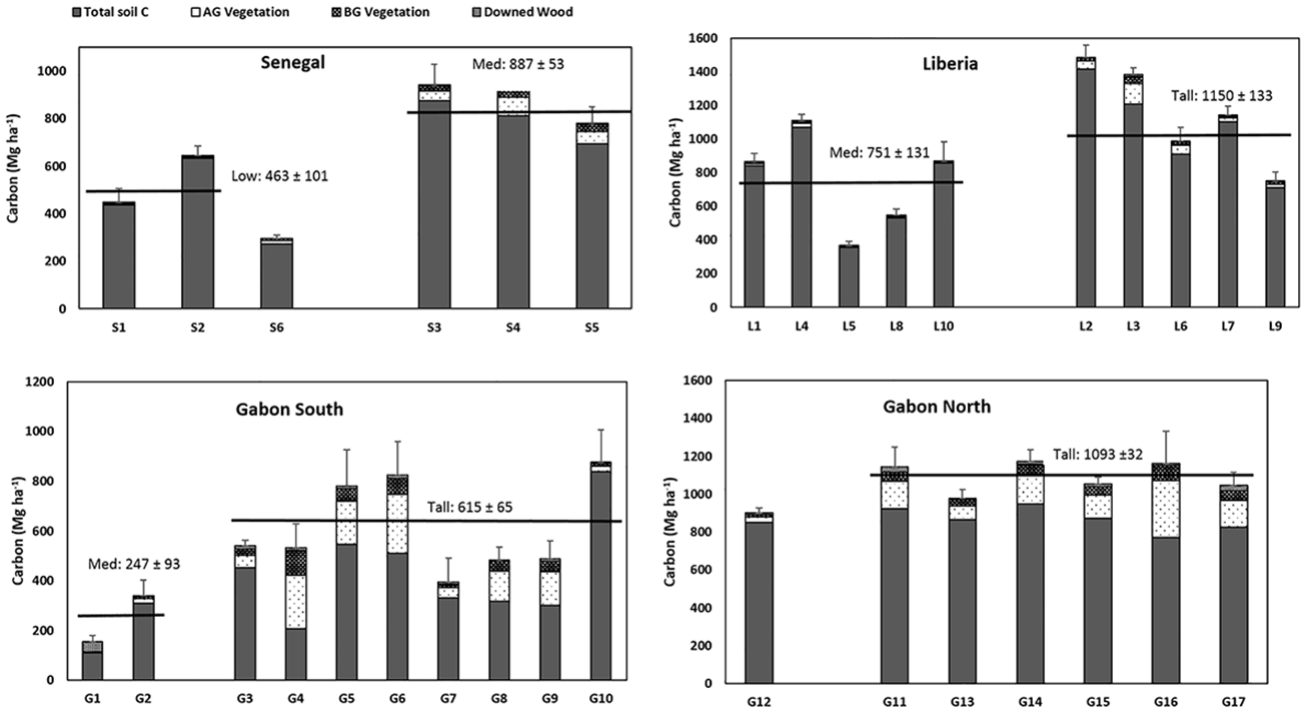
Ecosystem carbon stocks (Mg C ha^-1^) of sampled mangroves of West-Central Africa. Horizontal lines are the location means (± SE) of tall, medium or low mangroves.

Total aboveground carbon pools of the mangroves of West-Central Africa ranged from 5.2 to 312 Mg C ha^-1^ (Fig 5; S2 Table). Taller statured mangrove communities were significantly greater in aboveground carbon than lower statured communities at all sampling sites (p ≤ 0.06). There was a large variation in vegetation biomass even within structurally similar mangroves. For example, within the tall mangrove sites of Gabon South, aboveground carbon in the Lac Sounga Deux mangrove stand was 60 Mg C ha^-1^ compared to 325 Mg C ha^-1^ in the Mwana Mouele South stand (S2 Table).

Among the tall mangroves, the mean aboveground carbon ranged from 61 Mg C ha^-1^ in Liberia to 175 Mg C ha^-1^ in Gabon North (p = 0.01). The mean aboveground carbon of medium and low mangroves was < 59 Mg C ha^-1^. The mean TAGC pool of all sampled mangroves in this West-Central Africa study was 84 ± 14 Mg C ha^-1^. The majority of the aboveground pool was comprised of trees; dead wood was usually a minor component. This is similar to the cumulative default value of 82 Mg C ha^-1^ consisting of trees, downed wood and litter for mangrove aboveground carbon mass provided by the IPCC [17].

### Soil properties

Mean soil porewater salinity ranged from 64 ppt in the highly saline semiarid Senegal mangroves to 16 ppt in the tidal tropical monsonal Liberia sites. Pore water salinity at the Gabon sites was 30 ppt at Gabon North and 24 ppt at Gabon South (S1 Table). There was a narrow range of soil pH ranging from 6.4 in Gabon South to 7.1 in Gabon North. The soils of about 82% of the 33 sampled sites were ≥300 cm in depth; only 6 of the sampled sites had depths to bedrock or marine sands <300 cm.

There were distinct differences in the soil properties between the different vegetation types at the sites (Table 2). For example, at Senegal, the mean bulk density was higher and the nitrogen and carbon concentration were lower at all sampled soil depths in low mangroves compared to medium mangroves. Similar results were found comparing the medium to tall mangroves at Liberia. For example, the carbon concentration of surface soils was 7.0 and 14.6% in medium and tall mangroves, respectively. At depths of > 100 cm, the mean carbon concentration was 4.0% for medium and 9.7% tall mangroves.

**Table 2:** Soil bulk density (g cm^-3^), carbon concentration (%), nitrogen concentration (%) and carbon pools (Mg C ha^-1^) of sampled mangroves partitioned into low, medium and tall communities in Atlantic coastal mangroves of Africa.

Mangrove type	Soil depth (cm)	Bulk density (g cm^-3^)		Carbon concentration (%)		Nitrogen concentration (%)		C:N	Carbon storage* (Mg C ha^-1^)
		Range	Mean ± SE	Range	Mean ± SE	Range	Mean ± SE		Mean ± SE
**Senegal**									
**Low**	0–15	0.35–1.22	0.7 ± 0.07	0.97–11.06	5.5 ± 0.9	0.06–0.35	0.2 ± 0.02	28.0	41.1 ± 4.3
	15–30	0.31–1.25	0.8 ± 0.08	0.76–17.92	4.7 ± 1	0.04–0.53	0.2 ± 0.03	26.2	39.3 ± 4.6
	30–50	0.46–1.5	0.9 ± 0.09	0.44–9.09	3.4 ± 0.6	0.03–0.35	0.1 ± 0.02	24.4	45.2 ± 7.0
	50–100	0.46–1.57	1.0 ± 0.1	0.40–8.33	2.4 ± 0.5	0.02–0.28	0.1 ± 0.02	26.6	89.2 ± 11.4
	>100	0.45–1.53	1.1 ± 0.07	0.38–9.04	1.9 ± 0.5	0.02–0.29	0.1 ± 0.01	24.2	232.9 ± 30.9
**Medium**	0–15	0.20–1.56	0.6 ± 0.10	0.62–13.75	6.6 ± 1.0	0.02–0.52	0.3 ± 0.04	25.0	39.8 ± 3.4
	15–30	0.27–0.58	0.4 ± 0.02	4.95–14.35	8.9 ± 0.8	0.17–0.61	0.3 ± 0.03	27.9	48.5 ± 2.3
	30–50	0.29–0.70	0.5 ± 0.03	0.04–11.61	6.3 ± 0.6	0.18–0.51	0.3 ± 0.02	22.8	53.8 ± 4.3
	50–100	0.34–0.93	0.6 ± 0.03	0.53–9.12	4.7 ± 0.4	0.03–0.32	0.2 ± 0.01	26.3	122.6 ± 6.6
	>100	0.48–1.47	0.8 ± 0.07	0.99–6.33	3.7 ± 0.4	0.06–0.21	0.1 ± 0.01	27.1	529.3 ± 34.4
**Liberia**									
**Medium**	0–15	0.25–1.17	0.6 ± 0.05	1.09–14.82	7 ± 0.8	0.05–0.48	0.2 ± 0.03	29.0	44.3 ± 2.6
	15–30	0.22–1.06	0.5 ± 0.04	1.6–22.5	9.2 ± 0.9	0.06–0.57	0.3 ± 0.03	35.4	57 ± 2.4
	30–50	0.21–1.30	0.6 ± 0.05	0.53–18.77	8.4 ± 1.0	0.02–0.60	0.2 ± 0.03	37.7	72.1 ± 4.1
	50–100	0.20–1.36	0.7 ± 0.05	0.67–14.49	5.8 ± 0.6	0.02–0.42	0.2 ± 0.02	35.8	154.2 ± 9.3
	>100	0.23–1.43	0.9 ± 0.07	0.53–12.58	4.0 ± 0.7	0.02–0.55	0.2 ± 0.03	26.7	404.0 ± 126.0
**Tall**	0–15	0.18–0.61	0.3 ± 0.02	3.33–23.91	14.6 ± 1.1	0.17–0.84	0.5 ± 0.04	28.6	53.6 ± 2.3
	15–30	0.13–0.72	0.3 ± 0.03	3.46–31.35	12.9 ± 1.4	0.13–0.76	0.4 ± 0.03	30.3	50.8 ± 3.1
	30–50	0.13–0.86	0.3 ± 0.03	1.35–28.6	14.2 ± 1.5	0.08–0.74	0.4 ± 0.03	34.0	66.3 ± 4.4
	50–100	0.12–0.82	0.3 ± 0.03	3.19–41.15	14.3 ± 1.5	0.13–0.68	0.4 ± 0.03	36.4	185.6 ± 9.8
	>100	0.16–1.25	0.5 ± 0.05	1.34–26.74	9.7 ± 1.1	0.06–0.71	0.3 ± 0.03	30.7	714.0± 91.0
**Gabon North**									
**Medium**	0–15	0.24–0.29	0.3 ± 0.01	14.01–17.90	16.0 ± 0.7	0.47–0.62	0.6 ± 0.02	29.1	63.7 ± 4.4
	15–30	0.22–0.35	0.3 ± 0.02	12.66–21.55	16.9 ± 1.5	0.44–0.54	0.5 ± 0.01	33.6	65.8 ± 2.9
	30–50	0.21–0.33	0.3 ± 0.02	12.77–16.86	14.6 ± 0.8	0.43–0.52	0.5 ± 0.01	31.2	76.5 ± 8.2
	50–100	0.25–0.39	0.3 ± 0.02	8.27–19.46	12.1 ± 1.6	0.33–0.44	0.4 ± 0.02	31.4	195.4 ± 23.3
	>100	0.37–0.58	0.5 ± 0.03	3.39–5.97	4.8 ± 0.4	0.13–0.27	0.2 ± 0.02	23.8	451.0 ± 19.0
**Tall**	0–15	0.17–0.46	0.3 ± 0.01	7.09–24.04	16.4 ± 0.8	0.34–0.81	0.6 ± 0.02	28.7	64.5 ± 1.9
	15–30	0.16–0.49	0.3 ± 0.01	5.42–22.32	14.8 ± 0.7	0.29–0.66	0.5 ± 0.01	30.3	52.3 ± 2.2
	30–50	0.19–0.54	0.3 ± 0.01	5.64–18.67	12.3 ± 0.5	0.29–0.63	0.4 ± 0.01	28.7	66.3 ± 2.3
	50–100	0.23–0.48	0.4 ± 0.01	4.90–14.94	8.6 ± 0.4	0.26–0.57	0.4 ± 0.01	24.5	152.5 ± 5.7
	>100	0.32–0.56	0.4 ± 0.01	4.42–12.86	6.3 ± 0.2	0.24–0.49	0.3 ± 0.01	21.7	532.0 ± 22.0
**Gabon South**									
**Medium**	0–15	0.21–1.32	0.8 ± 0.1	1.32–17.8	5.1 ± 1.3	0.06–0.76	0.2 ± 0.06	22.3	40.3 ± 3.6
	15–30	0.41–1.73	1.1 ± 0.11	0.41–13.28	2.3 ± 1.0	0.03–0.2	0.1 ± 0.01	33.3	26.1 ± 6.7
	30–50	0.94–1.66	1.4 ± 0.07	0.20–5.56	1.1 ± 0.4	0.05–0.2	0.1 ± 0.02	12.4	25.8 ± 7.4
	50–100	0.91–1.93	1.5 ± 0.09	0.09–2.42	0.6 ± 0.2	0.08–0.12	0.1 ± 0.01	5.7	36.4 ± 9.8
	>100#	1.52	1.5	0.64	0.6	nd	nd	nd	80.1 ± 69.0
**Tall**	0–15	0.09–1.46	0.6 ± 0.06	0.63–36.89	12.3 ± 1.6	0.04–1.44	0.5 ± 0.06	22.6	51.3 ± 4.8
	15–30	0.11–1.55	0.7 ± 0.07	0.24–34.88	7.1 ± 1.3	0.04–1.16	0.4 ± 0.05	19.9	45.9 ± 11.9
	30–50	0.14–1.63	0.9 ± 0.07	0.14–27.81	4.9 ± 0.9	0.05–1.14	0.3 ± 0.04	17.8	41.2 ± 3.5
	50–100	0.17–1.73	1.2 ± 0.05	0.09–5.76	1.4 ± 0.2	0.08–0.26	0.1 ± 0.01	11.3	68.6 ± 8.2
	>100	0.14–1.63	1.3 ± 0.06	0.11–8.44	1.4 ± 0.3	0.09–0.32	0.2 ± 0.02	7.2	231.0 ± 47.0

^#^ Only one site from this depth hence no range and no SE. nd = no data collected.

While taller statured mangroves tend to have higher soil nitrogen and carbon concentrations, there were large differences in soil properties and patterns of soil carbon storage between the tall mangroves of Liberia, Gabon North and Gabon South (Table 2). All tall mangroves tended to have relatively high carbon concentrations in the surface soils (>12%). Further, the carbon concentrations tended to remain high throughout the soil profile at the Liberia and Gabon North tall mangroves with mean concentrations of 6.3 to 9.7% at depths of >100 cm. In contrast, soil carbon concentration of the Gabon South tall mangroves was 1.4% at all soil depths >50 cm. The soil bulk density of the Gabon South tall mangroves was 2–3 fold higher than similar depths in Liberia and Gabon North tall mangroves (e.g., mean soil bulk density at depths of >100 cm was ≤0.50 g cm^3^ in Liberia and Gabon North and 1.30 g cm^3^ at Gabon South). The higher bulk density reflects the coarse sand texture comprising the soil horizons at Gabon South compared to finer textured sediments that characterized the soils at the other locations.

Differences in the soil bulk density and carbon concentration resulted in similar differences in soil carbon pools when comparing tall mangroves between the regions. While mean soil carbon pools at depths <50 cm were similar among all tall mangrove locations, there was significantly less soil carbon at depths >50 cm at the Gabon South site. Soil carbon pools at the depth of 100–300 cm was 714 and 532 Mg C ha^-1^ at Liberia and Gabon North, respectively, but 231 Mg C ha^-1^ at Gabon South.

There were differences in soil carbon storage between low and medium mangroves in Senegal and between medium and tall mangroves at the other locations (Table 2). The most pronounced differences were at depths >50 cm. For example, in Senegal, the total soil carbon storage at soil depths >50 cm was 322 and 652 Mg C ha^-1^ for low and medium mangroves, respectively. In Liberia, carbon pools >50 cm were 558 Mg C ha^-1^ in the medium mangroves and 900 Mg C ha^-1^ in the tall mangroves (Table 2).

### Carbon stocks

The mean total ecosystem carbon stock was 799 ± 64 Mg C ha^-1^ for all of the mangroves sampled in this study (Table 3). Soils comprised an average of 86% of the total carbon stock. At the regional level, the lowest average ecosystem stocks were measured in Gabon South 539 ± 39 Mg C ha^-1^; n = 10) and the largest were measured in Gabon North (1063 ± 34 Mg C ha^-1^; n = 6) (Table 3). Among individual mangrove stands there was a high degree of variation in the ecosystem carbon stocks ranging from 154 Mg C ha^-1^ in a medium mangrove stand in Gabon South to 1382 Mg C ha^-1^ in a tall mangrove stand in Liberia (S2 Table).

**Table 3:** Ecosystem carbon stocks (Mg C ha^-1^) across the Western African landscape. Data are means ± one standard error.

Location	Total ecosystem	Downed wood	Vegetation	Soil (0–100 cm)	Total soils
**Senegal**	674 ± 46	1.2 ± 0.4	52 ± 18	240 ± 27	621 ± 98
**Liberia**	949 ± 47	4.9 ± 1.1	43 ± 15	342 ± 13	901 ± 137
**Gabon N**	1063 ± 34	17 ± 3.3	180 ± 40	345 ± 12	866 ± 46
**Gabon S**	539 ± 39	11.6 ± 3.6	136 ± 36	191 ± 31	392 ± 103
**All sites combined**	799 ± 64	10 ± 1	102 ± 11	278 ± 8	688 ± 59

The largest carbon stocks were found in the tall mangroves of Liberia and Gabon North with means >1093 Mg C ha^-1^ (Fig 5). There were significant differences between the different mangrove structural types; ecosystem carbon stocks were significantly greater in tall mangroves than medium mangroves at Liberia and Gabon South (p<0.05) and ecosystem carbon stocks were significantly greater in medium mangroves compared to low mangroves in Senegal (p = 0.05; Fig 5).

**Fig 6:**
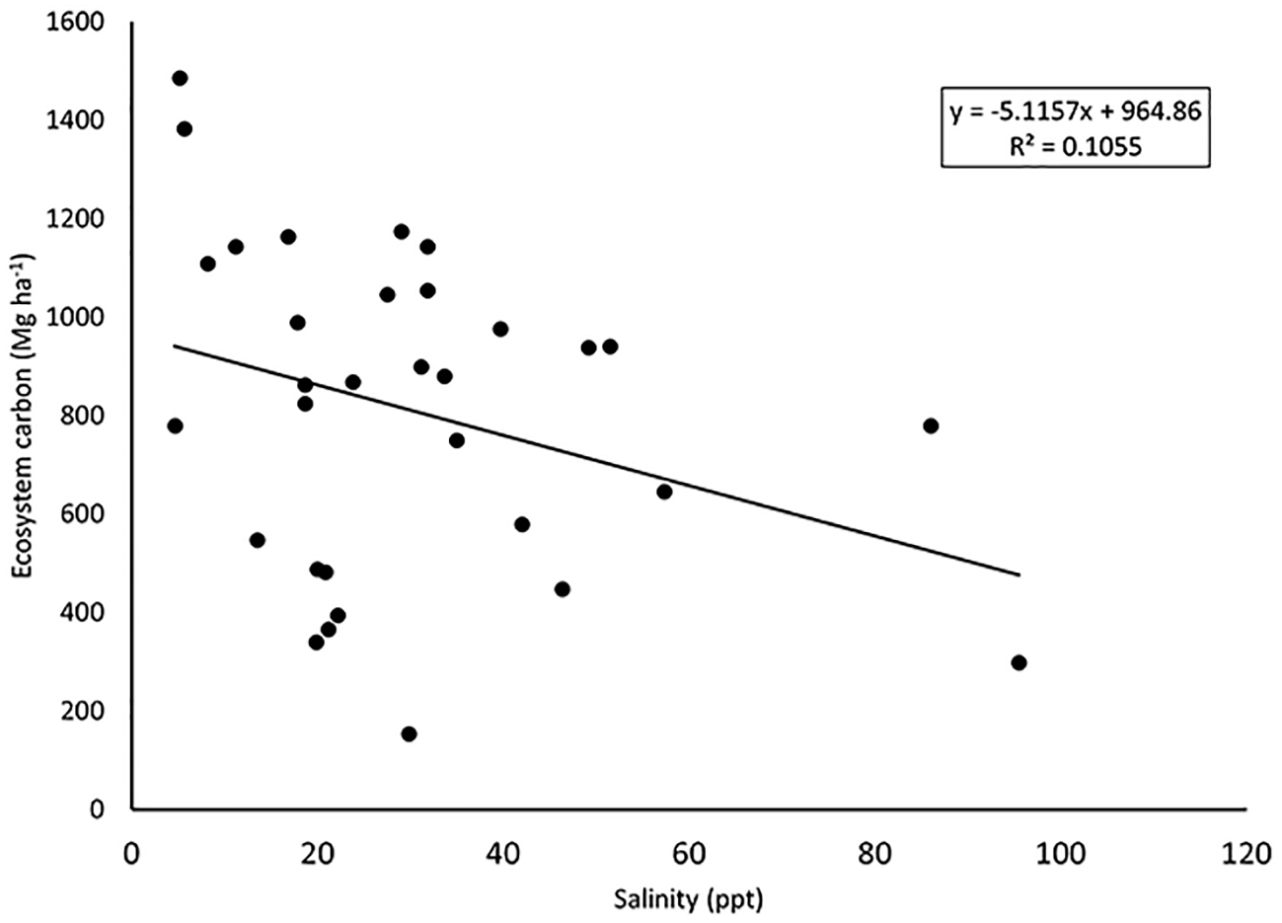
The relationship between total ecosystem carbon stocks and soil porewater salinity collected at the time when carbon stocks were measured.

While tall mangroves had greater ecosystem carbon stocks than adjacent medium mangroves, total aboveground carbon pools (live and dead vegetation) were poorly related to total ecosystem carbon stocks (r^2^ = 0.07). For example, the aboveground carbon mass of the tall mangroves of Gabon South was 133 Mg ha^-1^ while the ecosystem carbon stock totaled 615 Mg ha^-1^. In contrast, the aboveground carbon mass of the medium mangroves of Liberia South was 13 Mg ha^-1^ while the ecosystem carbon stock totaled 715 Mg ha^-1^ (Fig 5). Combining all sampled sites from this West-Central Africa study, ecosystem carbon stocks did not show a relationship to either soil salinity (r^2^ = 0.10) or soil pH (r^2^ = 0.04; Fig 6, S1 and S2 Tables).

Finally, there was great variation in the carbon stocks of tall mangroves across regions. The mean ecosystem carbon stock of tall mangroves at Gabon South (615 Mg C ha^-1^) was <56% of those of Gabon North or Liberia (>1093 Mg C ha^-1^; Fig 5). Differences were largely due to the significantly lower soil carbon pools in the coarse textured soils of Gabon South (Table 2).

### Comparisons to other global regions

**Fig 7:**
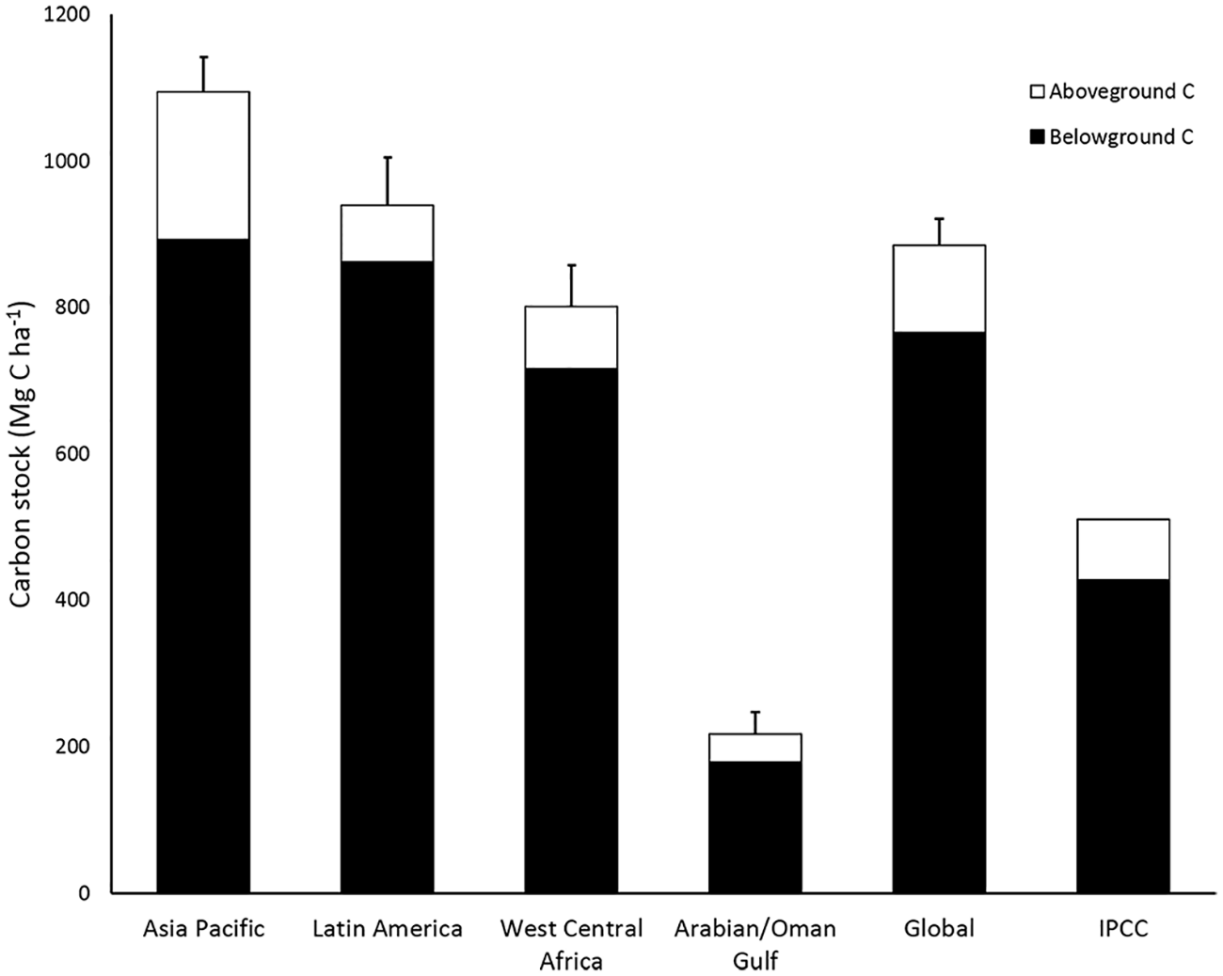
Carbon stocks of sampled mangroves in the Asia Pacific (n = 58), Latin America (n = 49), West Central Africa (n = 33), the United Arab Emirates (n = 18) and a global mean (n = 158). Data for Latin America are from [12, 22, 33, 34]. Data for the Asia Pacific are from [10, 12, 14, 30, 35]. Data from the Arabia/Oman Gulf are from [32]. Soils in all sites were sampled to bedrock, marine sands or to a depth of ≈300cm when sediments exceeded this depth (i.e., many deltas and estuaries). The IPCC values [17] include aboveground biomass from tropical regions (n = 72). Included in the aboveground carbon pools are dead wood and litter. The IPCC belowground carbon stocks include soils (N = 119) and estimates of belowground plant mass.

Carbon stocks of mangroves of West-Central Africa (799 Mg C ha^-1^) are slightly lower than those of Latin America (939 Mg C ha^-1^) or the Asia-Pacific (1,094 Mg C ha^-1^; Fig 7). They were much higher than Arabian/Oman Gulf mangroves found in extreme arid environments on coarse-textured soils (217 Mg C ha^-1^; [32]). In the analysis of the mangroves throughout the world, we found similarities common to all mangroves. For example, belowground carbon (roots and soils) comprised 82–91% of the total ecosystem carbon stock at all sites. This underscores the importance of including the soil carbon stock in climate change mitigation strategies.

## Discussion

Many studies have shown that variations in biomass and productivity can be attributed to differences in soil factors, such as nutrient availability, salinity or other stressors [36]. Mangroves along the Atlantic coast of Africa have a very broad ecological range of edaphic and climatic factors. For example, soil porewater salinity ranged from 5 to 96ppt (Table 1). Given effects of soil salinity on carbon cycling and allocation, we would expect that soil salinity would affect ecosystem carbon stocks. However, we found a weak relationship between salinity and both aboveground (r^2^ = 0.07) and total ecosystem carbon stocks (r^2^ = 0.10; Fig 6). These results may be influenced by the fact that we only measured soil porewater salinity once from these sampled sites. Therefore, this is unlikely a representation of the range of soil pore water salinity that is experienced by the vegetation on a seasonal basis. Additionally, other factors (e. g., soil texture and depth) likely have stronger controls on soil carbon storage. Examining the scatter in Fig 6 it is apparent that there are sites of low salinity with both high and low ecosystem carbon stocks.

At the site and regional levels, the larger statured communities typically had higher carbon stocks. For example, the mean ecosystem carbon stocks of all tall mangroves sampled in this study was 907 ± 73 Mg ha^-1^ compared to 710 ± 93 Mg ha^-1^ for medium mangroves (p = 0.05; S1 and S2 Tables). Within sampled regions, there were no significant differences in salinity when comparing between tall and medium, or medium and low mangroves. In addition, carbon stocks of the medium mangroves of the Saloum Delta, Senegal, with a relatively high soil salinity, were greater than the carbon stocks of tall mangroves of the Ndougou Lagoon, Gabon South with relatively low soil salinities (Table 1). Further, the higher latitude Senegal sites were surrounded by arid steppe as opposed to tropical forest/savanna mosaics in Gabon South where mean precipitation was almost three-fold greater. Finally, total ecosystem carbon stocks were poorly correlated to aboveground ecosystem carbon pools (r^2^ = 0.07). This underscores the importance and necessity to sample soil carbon pools to accurately ascertain ecosystem carbon stocks in mangroves. When quantifying carbon stocks, stratifying sites on the basis of geomorphic position and forest structure would facilitate improved accuracy of sampling [25, 37].

### Africa comparisons

Compared to other continents, African mangroves are among the least investigated with respect to ecosystem carbon stocks [3]. In Madagascar, Jones et al. [38] reported that ecosystem carbon stocks were 593 Mg C ha^-1^ in closed canopy and 367 Mg C ha^-1^ for open canopy mangroves. In this Madagascar study, soil carbon pools were limited to a depth of 1.5 m. In Tanzania, Stringer et al. [39] reported ecosystem stocks of mangroves ranged from 99 to 341 Mg C ha^-1^. Soils were sampled to a depth of 198 cm. Comparing East African studies to this study must also be tempered by the fact that composition and structure of mangroves of eastern Africa greatly differ from that of West-Central Africa [5].

Among the most important factors that determine the mass of the soil carbon pools in mangroves is soil carbon concentration. In this study the mean soil carbon concentration of all West-Central African soil samples was 8.6% (n = 979) and sample concentrations ranged from 0.04% to 41.5% (Table 2). At the regional level, mean carbon concentrations were 8.5% in Senegal, 10.1% in Liberia, 11.8% in Gabon North and 4.8% in Gabon South. In contrast, east African mangroves have been reported to have very low concentrations of carbon [37, 39]. Mean soil carbon content of mangrove soils in the Zambezi River Delta was 1.8% [36] and was 3.4% for mangroves soils of northern Madagascar [38].

Some of the differences in the soil carbon mass reported in the literature are reflective of the depths to which soil carbon pools were measured. Some studies limited carbon stock measurements to the top 1 to 2 m of soils compared to studies that sampled the soil profile to parent materials or at least the top 3 m [10, 33–34]. In alluvial and riverine mangroves and freshwater swamp forests where soils are usually quite deep (>3m), it has been found that soil properties, including carbon concentrations, are affected by land use at depths of 1 to 3 m [33–34]. Therefore, improvement of the accuracy of the determination of the influences of land use on ecosystem carbon dynamics requires measurements and reporting of the stocks and stock changes of the entire soil profile. In estuarine, deltaic and riverine landscapes with very deep soils, sampling and reporting carbon pools to depths of 3m would be most prudent to capture carbon pools vulnerable to loss via land use or climate change.

### Global comparisons

The global mean carbon stocks for mangroves based upon a total of 158 stands sampled was 885 Mg C ha^-1^. While the mean of the African mangroves was just below the global estimate, it is important to note the range of carbon stocks of the African mangroves of this study ranged from 154 to 1382 Mg C ha^-1^. Further, the structural diversity of these mangroves varied from low mangroves <2m height to tall mangroves >40m in height. This is a similar structural range encountered in the Latin American mangrove studies (e.g., refs [22, 33, 34]). Sites from the Asia Pacific in this example were all tall mangroves and were limited to areas of relatively high rainfall.

Globally, ecosystem carbon stocks of individual stands of mangroves ranged from 79 Mg C ha^-1^ (Arabian/Oman Gulf) to 2208 Mg C ha^-1^ (Indonesia). Mangroves > 2000 Mg C ha^-1^ were measured in Indonesia [14], Mexico [37], Honduras [31], and Micronesia [30]. Similar to Senegal, the mangroves of the Arabian/Oman Gulf were low and medium mangroves. While both of these regions are arid, the Senegal mangroves with larger ecosystem carbon stocks, were located in deltaic/estuarine environments compared to fringing mangroves of the Arabian/Oman Gulf.

The current IPCC Tier 1 default values for mangrove ecosystem carbon stocks is about 511 Mg C ha^-1^ ([17]; Fig 7) and belowground carbon comprises 84% of this estimate (428 Mg ha^-1^). The IPCC value is much lower than our results of mangrove ecosystem carbon stocks for Africa, the Indo-Pacific and Latin America. The IPCC estimate is 373 Mg C ha^-1^ lower or only 58% of our calculated global mean (Fig 7). Utilization of this estimate could result in significant underestimations in both the ecosystem carbon stocks and in the greenhouse gas emissions arising from land use change in mangroves. For example, in a study of land use change in mangroves, mean greenhouse gas emissions from conversion of mangrove to shrimp ponds and cattle pastures was 2033 Mg CO_2_e ha^-1^ [12]. This carbon loss (equivalent to 554 Mg C ha^-1^), exceeds the entire IPCC default value for ecosystem carbon stocks in mangroves [17]. The global carbon stocks estimate reported here (884 Mg C ha^-1^; Fig 7) is derived from a relatively large sample size and yields a more realistic Tier 1 value for the estimation of ecosystem carbon stocks than that provided by the IPCC [17]. The sample size for the IPCC default values was ≈119 for soils and ≈72 for vegetation [17]. Utilizing the ecosystem carbon stocks values of the mangroves in Fig 7 is suggested to be an improved estimation of default estimates (Tier 1 values) of mangroves for the Indo-Pacific, Latin America, and West Central Africa.

## Supporting information

S1 Table Characteristics of sampling locations in Liberia, Senegal, Gabon North and Gabon South. (PDF)

S2 Table Ecosystem carbon stocks in Liberia, Senegal and Gabon (north and south). Numbers are mean stocks ± one standard error.
(PDF)
