# Supplementary material for: Ecosystem carbon stocks of mangroves across broad environmental gradients in West-Central Africa: Global and regional comparisons
Source: PLoS One. 2017 Nov 13;12(11):e0187749. doi: 10.1371/journal.pone.0187749 (PMC5683642; doi:10.1371/journal.pone.0187749)
Supplement: S1 Table — (PDF) [file pone.0187749.s001.pdf]

S1 Table. Characteristics of sampling locations in Liberia, Senegal, Gabon North and Gabon South.

Site	Site number	Mangrove forest type	Dominant species	Latitude	Longitude	pH	Salinity (ppt)	Soil Depth (cm)
				**Liberia**				
**NCM1**	L1	Medium	*R. racemosa*	N 5°47.583'	W 9°58.216'	6.7	18.7	≥300
**NCT2**	L2	Tall	*R. racemosa*	N 5°48.236'	W 9°57.086'	6.9	5.2	≥300
**NCT3**	L3	Tall	*R. racemosa*	N 5°48.277'	W 9°57.274'	6.4	5.7	≥300
**NCM4**	L4	Medium	*R. racemosa*	N 5°48.069'	W 9°57.361'	6.6	8.2	≥300
**NCM5**	L5	Medium	*R. racemosa*	N 5°47.955'	W 9°58.242'	6.2	21.2	90
**NCT6**	L6	Tall	*R. racemosa*	N 5°47.571'	W 9°58.277'	7.3	17.8	≥300
**MRT7**	L7	Tall	*R. racemosa*	N 5°56.321'	W 10°05.089'	6.4	11.2	≥300
**MRM8**	L8	Medium	*R. racemosa*	N 5°55.747'	W 10°04.576'	6.9	13.5	≥300
**MRT9**	L9	Tall	*R. racemosa*	N 5°55.853'	W 10°04.417'	nd*	35.0	≥300
**BRM10**	L10	Medium	*R. racemosa*	N 5°54.992'	W 10°03.866'	6.8	23.8	220
				**Senegal**				
**Baouth**	S2	Low	*R. mangle*	N 13°57.725'	W 16°38.736'	6.8	46.3	≥300
**Diamniadio**	S3	Medium	*R. mangle*	N 14°03.157'	W 16°32.626'	6.3	57.3	≥300
**Djirnda**	S5	Medium	*R. racemosa*	N 14°04.536'	W 16°34.014'	6.4	51.5	≥300
**Fambine**	S4	Medium	*R. racemosa*	N 13°58.567'	W 16°36.945'	6.4	49.2	≥300
**Mounde**	S1	Low	*R. racemosa*	N 14°00.062'	W 16°34.230'	6.4	86.0	172
**Sang**	S6	Low	*A. nitida*	N 14°01.957'	W 16°34.713'	6.6	95.5	≥300

S1 Table (Continued)

Site	Site Number	Mangrove forest type	Dominant species	Latitude	Longitude	pH	Salinity (ppt)	Soil depth (cm)
				**Gabon South**				
**Jardin du Elephant**	G1	Medium	*R. racemosa*	S2°42.360'	E 9º54.438'	6.3	29.8	222
**Case Shell**	G2	Medium	*R. racemosa*	S2°29.455'	E 9º43.826'	6.7	19.8	182
**Mwana Mouele**	G3	Tall	*A. germinans*	S2°23.593'	E 9º38.960'	6.7	42.0	≥300
**Mwana Mouele S**	G4	Tall	*A. germinans*	S2°24.505'	E 9º38.861'	nd	nd	183
**Lac Simba Deux**	G5	Tall	*R. racemosa*	S2°27.742'	E 9º44.485'	6.2	4.7	≥300
**Lac Simba**	G6	Tall	*R. racemosa*	S 2°27.018'	E 9°44.757'	5.9	18.7	≥300
**Lac Sounga Deux**	G7	Tall	*R. racemosa*	S 2°26.927'	E 9°43.907'	6.8	22.2	≥300
**Lac Sounga**	G8	Tall	*R. racemosa*	S 2°25.997'	E 9°43.729'	6.2	20.8	≥300
**Paga**	G9	Tall	*R. racemosa*	S 2°30.210'	E 9°47.706'	6.7	20.0	≥300
**Ndougou**	G10	Tall	*R. racemosa*<br>*A. germinans*	S 2°25.019'	E 9°38.923'	6.7	33.7	≥300
				**Gabon North**				
**Nzeme**	G11	Tall	*R. racemosa*	N 00°30.261'	E 9°36.971'	7.3	31.8	≥300
**Mud bank**	G12	Medium	*R. racemosa*	N 00°34.422'	E 9°33.822'	7.4	31.2	≥300
**Moka**	G13	Tall	*R. racemosa*	N 00°36.209'	E 9°28.340'	6.9	39.7	≥300
**Moka River II**	G14	Tall	*R. racemosa*	N 00°37.049'	E 9°28.714'	7.4	29.0	≥300
**Bambouchine**	G15	Tall	*R. racemosa*	N 00°27.836'	E 9°36.015'	7.1	31.8	≥300
**Massotie**	G16	Tall	*R. racemosa*	N 00°41.708'	E 9°37.297'	6.9	16.8	≥300
**Nzeme River II**	G17	Tall	*R. racemosa*	N 00°28.458'	E 9°39.281'	6.9	27.5	≥300

*nd= no data due to equipment failures or other unforeseen difficulties in the field.
