# Supplementary material for: Ecosystem carbon stocks of mangroves across broad environmental gradients in West-Central Africa: Global and regional comparisons
Source: PLoS One. 2017 Nov 13;12(11):e0187749. doi: 10.1371/journal.pone.0187749 (PMC5683642; doi:10.1371/journal.pone.0187749)
Supplement: S2 Table — Numbers are mean stocks ± one standard error. (PDF) [file pone.0187749.s002.pdf]

S2 Table. Ecosystem carbon stocks in Liberia, Senegal and Gabon (north and south). Numbers are mean stocks ± one standard error.

	Carbon stocks (Mg C ha^{-1}) partitioned into various ecosystem pools					Fraction (%) of total ecosystem C		
**Location/sample site**	**Downed wood**	**Vegetation**	**Soil (0-100 cm)**	**Total soil carbon**	**Total ecosystem**	**Downed wood**	**Vegetation**	**Soils**
				**Liberia**				
**NCM1**	2.3 ± 0.6	16 ± 4	334 ± 23	843 ± 53	862 ± 53	0.3	1.9	97.8
**NCT2**	3.3 ± 0.9	64 ± 13	428 ± 17	1418 ± 75	1484 ± 73	0.2	4.3	95.6
**NCT3**	12.4 ± 3.2	162 ± 28	360 ± 36	1203 ± 43	1382 ± 44	0.9	11.7	87.0
**NCM4**	2.8 ± 0.5	36 ± 6	332 ± 14	1069 ± 39	1108 ± 38	0.3	3.2	96.5
**NCM5**	1.6 ± 0.6	6 ± 1	348 ± 25	358 ± 28	366 ± 29	0.4	1.6	97.8
**NCT6**	4.2 ± 1.3	75 ± 18	309 ± 31	910 ± 77	989 ± 80	0.4	7.6	92.0
**MRT7**	8.4 ± 0.5	30 ± 8	386 ± 19	1104 ± 58	1142 ± 51	0.7	2.6	96.7
**MRM8**	2.6 ± 0.5	7 ± 3	286 ± 19	530 ± 37	540 ± 39	0.5	1.3	98.1
**MRT9**	8 ± 3	30 ± 7	299 ± 12	710 ± 51	748 ± 54	1.1	4.0	94.9
**BRM10**	2.8 ± 0.8	5 ± 2	338 ± 29	858 ± 115	866 ± 116	0.3	0.6	99.1
				**Senegal**				
**Mounde**	0 ± 0	11 ± 2	278 ± 22	436 ± 60	448 ± 59	0.0	2.5	97.3
**Baouth**	0 ± 0	12 ± 2	255 ± 14	634 ± 39	646 ± 40	0.0	1.9	98.1
**Diamniadio**	0.8 ± 0.3	62 ± 25	257 ± 14	876 ± 68	940 ± 89	0.1	6.6	93.2
**Djirnda**	0.6 ± 0.2	122 ± 43	300 ± 12	811 ± 32	933 ± 46	0.1	13.1	86.9
**Fambine**	2.2 ± 0.4	83 ± 20	236 ± 22	694 ± 62	779 ± 71	0.3	10.7	89.1
**Sang**	0 ± 0	23 ± 6	111 ± 8	273 ± 15	296 ± 15	0.0	7.8	92.2

S2 Table (Continued)

Sample site	Carbon stocks ($Mg\ C\ ha^{-1}$) partitioned into various ecosystem pools					Fraction (%) of total ecosystem C		
	Downed wood	Vegetation	Soil (0-100 cm)	Total soil carbon	Total ecosystem	Downed wood	Vegetation	Soils
**Gabon North**								
**Nzeme**	28.5 ± 9.9	187 ± 35	324 ± 22	924 ± 79	1140 ± 107	2.5	16.4	81.1
**Mud bank**	7.5 ± 1	36 ± 5	401 ± 19	852 ± 29	896 ± 28	0.8	4.0	95.1
**Moka**	7.6 ± 2.7	104 ± 19	324 ± 16	864 ± 35	975 ± 47	0.8	10.7	88.6
**Moka River II**	17.2 ± 5.8	206 ± 80	371 ± 26	949 ± 33	1172 ± 59	1.5	17.6	81.0
**Bambouchine**	16.9 ± 8.8	164 ± 40	333 ± 16	872 ± 20	1053 ± 34	1.6	15.6	82.8
**Massotie**	12.6 ± 6.9	380 ± 160	315 ± 21	770 ± 41	1163 ± 170	1.1	32.7	66.2
**Nzeme River II**	28.8 ± 6.7	185 ± 40	346 ± 15	827 ± 43	1041 ± 68	2.8	17.8	79.4
**Gabon South**								
**Jardin du Elephant**	41.7 ± 26.7	3 ± 1	98 ± 9	109 ± 16	154 ± 26	27.1	1.9	70.8
**Case Shell**	2 ± 0.9	25 ± 11	159 ± 39	308 ± 53	335 ± 64	0.6	7.5	91.9
**Mwana Mouele**	13.5 ± 4.4	75 ± 10	88 ± 17	452 ± 17	541 ± 22	2.5	13.9	83.5
**Mwana Mouele South**	11.4 ± 2	314 ± 100	57 ± 9	208 ± 34	533 ± 95	2.1	58.9	39.0
**Lac Simba Deux**	9.7 ± 5.4	221 ± 120	264 ± 45	547 ± 102	778 ± 147	1.2	28.4	70.3
**Lac Simba**	13.2 ± 3.6	301 ± 65	236 ± 35	511 ± 88	825 ± 135	1.6	36.5	61.9
**Lac Sounga Deux**	6.9 ± 3	53 ± 21	177 ± 39	332 ± 90	392 ± 96	1.8	13.5	84.7
**Lac Sounga**	5.6 ± 2.9	156 ± 38	225 ± 15	318 ± 23	480 ± 53	1.2	32.5	66.3
**Paga**	10.6 ± 6.5	173 ± 88	219 ± 17	300 ± 20	484 ± 72	2.2	35.7	62.0
**Ndougou**	1.2 ± 0.3	34 ± 11	389 ± 85	838 ± 129	872 ± 132	0.1	3.9	96.1
